# Population heterogeneity in *Cryptococcus neoformans*: Impact on pathogenesis

**DOI:** 10.1371/journal.ppat.1012332

**Published:** 2024-07-11

**Authors:** Ruchi Agrawal, Raffael J. Araújo de Castro, Aude Sturny-Leclère, Alexandre Alanio

**Affiliations:** 1 Institut Pasteur, Université Paris Cité, National Reference Center for Invasive Mycoses and Antifungals, Translational Mycology Group, Mycology Department, Paris, France; 2 Laboratory of Applied Immunology, Department of Cell Biology, Institute of Biological Sciences, University of Brasilia, Brasília, Distrito Federal, Brazil; 3 Mycology-parasitology Laboratory, Hôpital Saint-Louis AP-HP, Paris, France; University of Maryland, Baltimore, UNITED STATES

## Introduction

*Cryptococcus neoformans* was first isolated from a patient in 1894 [1] and 130 years later, it remains a prevalent global threat, especially for people living with human immunodeficiency virus (HIV) [2]. Human–*Cryptococcus* interaction begins in childhood, after inhalation of basidiospores which are widespread in the environment. Typically, this interaction advances in one of the 4 major possible ways: (I) latent/dormant (asymptomatic) cryptococcosis, where *C*. *neoformans* can hide in the host for years without any clinical symptoms; (II) pulmonary cryptococcosis (cryptococcal pneumonia), which may or may not need medical intervention depending on host’s underlying conditions; (III) disseminated cryptococcosis, where *C*. *neoformans* probably disseminates from lungs to different organs such as kidneys, bones, skin, and to the central nervous system by crossing the blood–brain barrier, after reactivation from dormancy; (IV) cryptococcal relapse, where the formerly treated infection resurfaces and requires specific medical management. Population heterogeneity (also referred to as phenotypic heterogeneity) provides a functional advantage to many microbial pathogens to survive in fluctuating conditions (please refer to this excellent review [3]). It can be defined as the preexisting diversity within an isogenic population. Population heterogeneity arises due to the phenotypic differences between individual cells in an otherwise genetically identical/homogenous population. Prominent examples of phenotypic heterogeneity (well known in bacteria but not much in fungi) are biofilm, antimicrobial persistence, heteroresistance, and cellular dormancy, which is often observed as a viable but nonculturable (VBNC) phenotype. As described further in the review, population heterogeneity provides benefits to the *C*. *neoformans* population in coping with unpredictable environmental and host conditions. It contributes to the survival of *C*. *neoformans* in challenging and ever-changing conditions such as switching from the outer atmosphere to the host lungs, from extracellular to intracellular niche (e.g., inside macrophages), from lungs to brain or other tissues, from compromised immune system to exposure to antifungal drugs.

**Fig 1:**
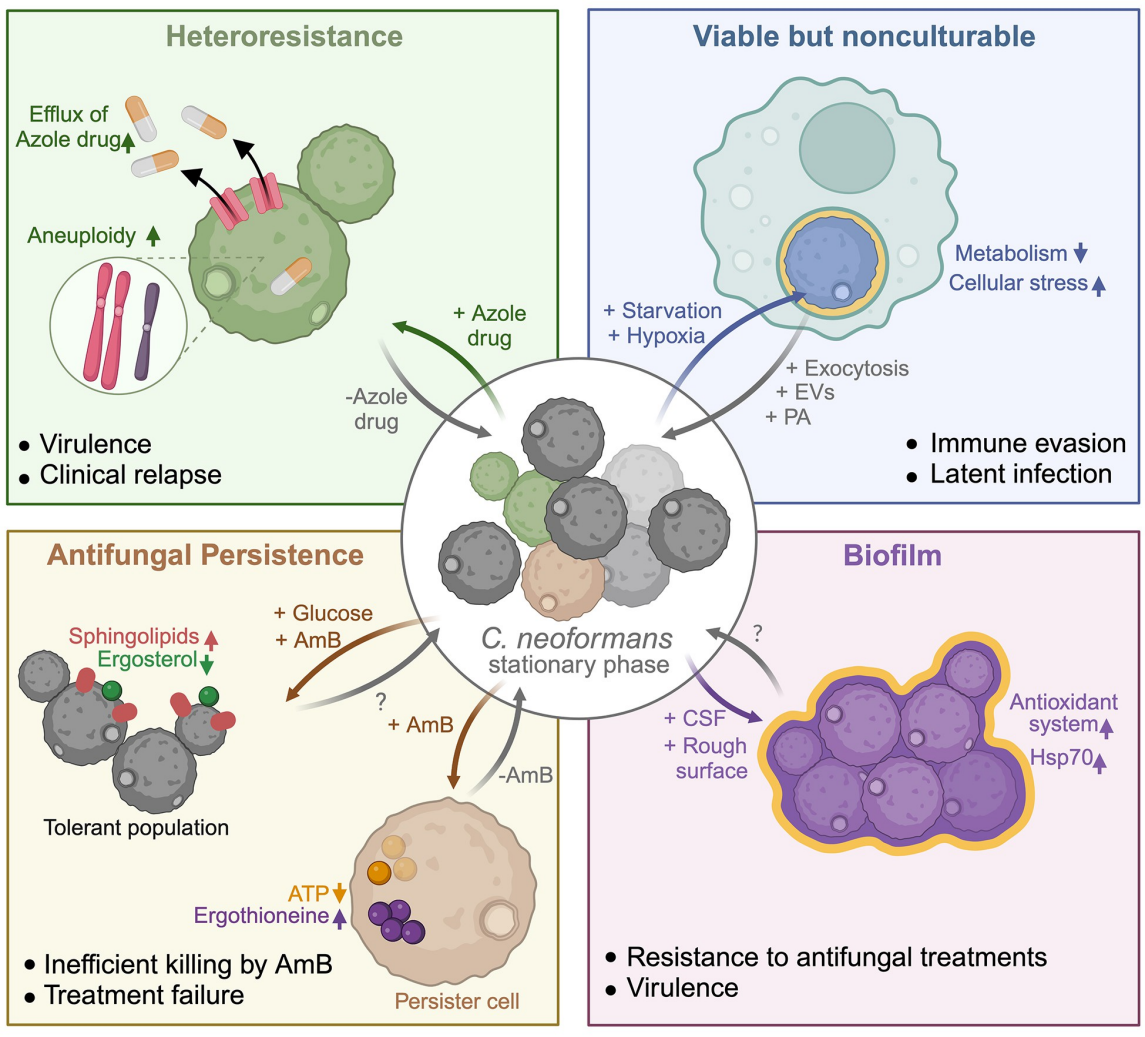
Population heterogeneity in *Cryptococcus neoformans* contributes to pathogenesis. *C*. *neoformans* displays phenotypic heterogeneity, which can either be intrinsic or generated in response to stress and environmental/host cues. These phenotypes are associated with diverse outcomes (mentioned in the bullet list), leading to enhanced pathogenicity and survival in the host. EVs, extracellular vesicles; PA, pantothenic acid; CSF, cerebrospinal fluid; AmB, Amphotericin B. Please refer to the text for details. Adapted from “Fate of Pristine Biochar in Soil,” by BioRender.com (2024). Retrieved from https://app.biorender.com/biorender-templates.

In this minireview, we discuss population heterogeneity manifested through heteroresistance, VBNC, persistence, and biofilm phenotypes. We review the influence of population heterogeneity on the survival and pathogenesis of *C*. *neoformans* (Fig 1).

## Heteroresistance in *C*. *neoformans* influences clinical relapse

Heteroresistance in *C*. *neoformans* was discovered in 1999 by Mondon and colleagues [4]. During heteroresistance, a small subpopulation of *C*. *neoformans* gains transient resistance to specific antifungal drugs, and when the drug pressure is removed, cells can revert to the original phenotype. It is a universal phenomenon and intrinsically present among all cryptococcal strains regardless of previous drug exposure. However, strains that show higher tolerance to fluconazole, exhibit an increased tendency of heteroresistance [5]. Intriguingly, strains with higher heteroresistance to fluconazole showed increased virulence in mice models [4,5]. Recently, Stone and colleagues studied heteroresistance in a cohort of 20 patients with HIV-associated cryptococcal meningitis [6]. They observed heteroresistance in *C*. *neoformans* populations of all the patients before starting the treatment, confirming that it is indeed inherent [6]. Further, studies performed in humans, mice, and in vitro models show that chromosomal aneuploidy is instrumental for heteroresistance. Often, chromosome 1 (chr1) is the first chromosome that gets affected by aneuploidy, followed by other chromosomes, depending on the severity of antifungal exposure [6,7]. The *ERG11* gene (encoding lanosterol 14α-demethylase, the target of azoles drug) and the *AFR1* gene (encoding an ATP-binding cassette (ABC)-type efflux pump to pump out azole drugs) are located on chr1 and are prominently up-regulated during chromosomal diploidy. However, heteroresistance is probably the result of multiple genes operating together, as disruption of individual genes showed no/very minor effect on heteroresistance [5]. Interestingly, Stone and colleagues found a significant increase in the proportion of heteroresistant subpopulations in patients receiving fluconazole monotherapy. Whereas, during the combination therapy with fluconazole and 5-fluorocytosine, the heteroresistance completely disappeared in 2 weeks [6]. As evident, heteroresistance could be one of the contributing factors in the relapse of cryptococcosis during fluconazole maintenance therapy; however, concrete epidemiological data is still lacking. So far, heteroresistance has only been shown with the “azole” class of antifungal drugs, hence, the question arises if it occurs with other classes of antifungal drugs. Moreover, studies are needed to understand the molecular mechanisms behind the differential heteroresistance in the strains with varied fluconazole sensitivity. Within aneuploidy, the driving mechanisms at transcriptional and translational levels, and besides aneuploidy, the role of other epigenetic changes such as heterochromatin formation, are interesting topics to be explored.

## Viable but nonculturable state in *C*. *neoformans* contributes to low-immunostimulatory capacity

Extensive epidemiological research established that dormant *C*. *neoformans* cells linger inside the host for years [8,9]. When recovered from macrophages or the lungs of infected mice, a tiny subpopulation of cryptococci exhibited lower metabolic activity than the rest of the population [10]. During resuscitation, this dormant subpopulation showed a significant delay in growth and needed certain molecules to revive [10]. Subsequently, a protocol inspired by the “Wayne model of hypoxia for mycobacterial dormancy,” was developed to generate dormant cryptococcal cells in vitro [11,12]. The protocol specifically generated cells called viable but nonculturable (VBNC) cells. In the VBNC state, cryptococci show very low metabolic activity and are unable to divide. However, they can become culturable after resuscitation, which mostly requires specific growth factors. A phenotypic microarray using 761 metabolites confirmed that these VBNC cells had lower metabolic capacity. These cells needed a specific growth factor, pantothenic acid, to efficiently exit from dormancy and resume growth. VBNC yeasts showed differential gene expression mainly in fatty acid metabolism pathways, signal transduction, and proton transport pathways [11]. Cellular stress pathways were up-regulated in VBNC yeasts, as seen by accumulated stress granules and poly(A) binding protein in the cytoplasm and nucleus, respectively [13]. Moreover, these yeasts showed a minimal immunostimulatory profile compared to proliferating or stationary phase cells in transcriptomics and cytokine profiling [14]. VBNC cells also displayed a decreased propensity to exit phagocytes [14]. Presumably, these traits facilitate them to hide inside the macrophages without getting noticed by the immune system, hence prolonging their survival. Later, non-lytic exocytosis of VBNC cells, and exposure to host-derived extracellular vesicles, allows the reactivation of VBNCs mainly in M0 and M2 macrophages [14]. Remarkably, a recent clinical study detected VBNC cells in cerebrospinal fluid (CSF) of patients undergoing therapy for cryptococcal meningitis [15]. Finding these cells in patients’ CSF even after induction therapy indicates that the VBNC state could be protecting the yeasts from antifungal treatments; however, this demands detailed research. More research is also needed to understand how VBNC phenotype differs among clinical strains and whether is it associated with virulence. The development of high-throughput assays appears necessary to test several environmental and host signals, which potentially influence the VBNC phenotype.

## Antifungal persistence in *C*. *neoformans* reduces the efficacy of current drug treatments

Although a well-known phenotype in bacteria since 1944 [16], persistence has only recently been investigated in *C*. *neoformans* by Wang and colleagues [17]. Persister cells are a small subpopulation of cells that remain viable in an otherwise lethal drug concentration. Following regrowth in drug-free media, the cells revert to the original drug-sensitive population which again contains a small subpopulation of persister cells; whereas tolerance is the ability of the entire cryptococcal population to survive the drug treatment. Using classical time-kill assays, the authors found that some cells from the stationary phase culture of *C*. *neoformans* survived 24 h of fungicidal drug Amphotericin B (AmB) treatment [17]. Persistence against AmB in those experimental settings involved 2 parallel but independently operating mechanisms inside cryptococcal cells: (I) induction of antioxidant defense system (via ergothioneine production); and (II) reduction of energy metabolism (via ATP depletion). Further, using a murine model of pulmonary cryptococcosis, they validated that persister cells are also present in the lungs of infected mice. Intriguingly, an antidepressant, sertraline, showed some preliminary activity against these persister cells in vitro and in vivo [17], which needs further investigation. Another recent study from the same group explored the role of host-derived factors during cryptococcal antifungal tolerance. By screening a library of 340 metabolites, the authors identified that *C*. *neoformans* tolerance to AmB increases in high-glucose media, as more cells survived AmB treatment in the presence of high glucose in vitro [18]. Similarly, AmB was more lethal against *C*. *neoformans* in glucose-depleted human CSF. Transcriptomics and lipidomics approaches revealed that high glucose leads to low levels of ergosterol and high levels of sphingolipids in fungal cell membranes through fungal *MIG1* (zinc finger transcription factor for glucose repression pathway). This results in fewer target molecules (ergosterol) available in the membranes for AmB targeting. The mutant strain lacking the *MIG1* gene showed significantly reduced fungal load after AmB treatment, in the brains of mice with cryptococcal meningitis, confirming that brain glucose induces tolerance against AmB in vivo as well. Interestingly, a combination of AmB + aureobasidin A (sphingolipid biosynthesis inhibitor) showed better outcomes than the currently recommended combination treatment AmB+ flucytosine, in infected mice [18]. Although more research is needed, these 2 studies are great examples of the promises that adjuvant therapy holds. It is fascinating that basic parameters such as blood sugar fluctuations (attention to diabetes) could potentially generate variability in treatment outcomes. Perhaps, adjuvant therapy combined with strict dietary recommendations should be the area of future exploration. In terms of molecular biology, what are the other host factors besides glucose that potentiate/diminish antifungal persistence; is this phenomenon common to all clinical strains; or are there clinical strains that are genetically/epigenetically favored to develop persistence; these are some of the prospective subjects.

## Biofilm in *C*. *neoformans* promotes survival and virulence inside the host

Cryptococcal biofilm was first discovered in 1986 in a patient with ventriculoatrial shunt [19]. However, it was much later in 2005 that research started emerging on cryptococcal biofilm. In a biofilm, metabolically active *C*. *neoformans* cells are interwoven with extracellular polysaccharide material in a highly organized structure. Biofilm not only protects *C*. *neoformans* from multiple stresses such as heat, cold, and ultraviolet radiation, but cells in biofilm are also more resistant to antifungal treatment [20,21]. Interestingly, phagocytosis and subsequent killing by macrophages become difficult when *C*. *neoformans* is in a biofilm state [22]. In *Galleria mellonella*, cryptococcal biofilm showed more virulence, as cells from the biofilm caused more larvae death than planktonic cells [23]. A comparative proteomic study performed by Santi and colleagues revealed many interesting features of cryptococcal biofilm. Even though biofilm showed an overall decrease in proteins involved in general transcription and translation compared to planktonic cells, it was better prepared to combat cellular and oxidative stress, as seen by the abundance of these proteins in biofilm [24]. Notably, the Hsp70 protein, which is often found elevated in the sera of patients with pulmonary cryptococcosis [25], was also significantly high in biofilm. Thus, it seems likely that *C*. *neoformans* could form biofilm inside the human host, especially in the brain, as soap-bubble lesions or biofilm-like structures have been seen in infected mice brains [26]. However, clinical studies are needed to confirm cryptococcal biofilm inside the human host. Investigations are also required to compare the biofilm formation abilities of various clinical strains and to understand the correlation between biofilm and virulence/ pathogenesis. Furthermore, what factors influence the formation of biofilm and whether host-derived metabolites are involved, are still questions to be answered. Systematic studies are also needed to find the biomarkers of cryptococcal biofilm to carefully distinguish it from mere clumps of cells.
